# Getting in Front of Chronic Wasting Disease: Model-Informed Proactive Approach for Managing an Emerging Wildlife Disease

**DOI:** 10.3389/fvets.2020.608235

**Published:** 2021-01-06

**Authors:** Aniruddha V. Belsare, Joshua J. Millspaugh, J. R. Mason, Jason Sumners, Hildegunn Viljugrein, Atle Mysterud

**Affiliations:** ^1^Department of Fisheries and Wildlife, Boone and Crockett Quantitative Wildlife Center, Michigan State University, East Lansing, MI, United States; ^2^W.A. Franke College of Forestry and Conservation, Wildlife Biology Program, University of Montana, Missoula, MT, United States; ^3^Michigan Department of Natural Resources Executive in Residence, College of Agriculture and Natural Resources, Michigan State University, East Lansing, MI, United States; ^4^Missouri Department of Conservation, Columbia, MO, United States; ^5^Norwegian Veterinary Institute, Oslo, Norway; ^6^Department of Biosciences, Centre for Ecological and Evolutionary Synthesis (CEES), University of Oslo, Oslo, Norway

**Keywords:** agent-based models, chronic wasting disease, pre-emptive strategies, surveillance, white-tailed deer

## Abstract

Continuing geographic spread of chronic wasting disease (CWD) poses a serious threat to the sustainable future of cervids and hunting in North America. Moreover, CWD has been detected in captive cervids in South Korea and, in recent years, in free-ranging reindeer in Europe (Norway). Management of this disease is limited by logistical, financial, and sociopolitical considerations, and current strategies primarily focus on reducing host densities through hunter harvest and targeted culling. The success of such strategies in mitigating the spread and prevalence of CWD only upon detection is questionable. Here, we propose a proactive approach that emphasizes pre-emptive management through purposeful integration of virtual experiments (simulating alternate interventions as model scenarios) with the aim of evaluating their effectiveness. Here, we have used a published agent-based model that links white-tailed deer demography and behavior with CWD transmission dynamics to first derive a CWD outbreak trajectory and then use the trajectory to highlight issues associated with different phases of the CWD outbreak (pre-establishment/transition/endemic). Specifically, we highlight the practical constraints on surveillance in the pre-establishment phase and recommend that agencies use a realistic detection threshold for their CWD surveillance programs. We further demonstrate that many disease introductions are “dead ends” not leading to a full epidemic due to high stochasticity and harvesting in the pre-establishment phase of CWD. Model evaluated pre-emptive (pre-detection) harvest strategies could increase the resilience of the deer population to CWD spread and establishment. We conclude it is important to adaptively position CWD management ahead of, rather than behind, the CWD front.

## Introduction

Chronic wasting disease (CWD) is an emerging prion disease of North American cervid populations (including white-tailed deer *Odocoileus virginianus*, mule deer *Odocoileus hemionus*, and elk *Cervus canadensis*) that has been detected in free-ranging and captive cervids in 26 U.S. states and three Canadian provinces as well as in free-ranging reindeer *Rangifer tarandus* in Norway and in captive cervids in South Korea. The seemingly inexorable spread of CWD presents both short- and long-term threat to free-ranging cervids and to wildlife conservation in general. Field data show that CWD can markedly reduce cervid populations (1, 2); while modeling studies suggest that CWD may even cause extirpation of local cervid populations (3). As well, CWD-driven declines in hunting license revenue, and the unsustainable cost of existing CWD surveillance and management programs have obvious economic implications (4, 5). Deer and deer hunting underpin the economic well-being of North American conservation as deer hunting is an important source of revenue for wildlife related work and added conservation value through the protection and management of private lands that benefit other wildlife (6, 7).

The management of wildlife diseases in general and CWD in particular is constrained by limited scientific knowledge about transmission dynamics (8). Moreover, uncertainties about the current status of infection in many populations and the likelihood of success of different management actions are major challenges. These challenges are compounded by the controversial nature of deer management and popular and political opposition to disease management interventions (9, 10). Regardless, affected publics and politicians expect and demand that wildlife agencies respond promptly and effectively to disease threats. An effective approach to addressing uncertainties while dealing with complex systems is through the integration of formal models with management and related policy decision making (11). Modeling presents an economical and time sensitive alternative to direct field tests that are costly, require years to complete and are difficult to sustain. Such virtual explorations can enhance scientific understanding of complex systems and can be developed specifically for evaluating the utility of various interventions and regulatory packages.

There are many different ways to model CWD dynamics depending on the objectives (12). Here, we build our argument using a published agent-based modeling framework, *Ov*CWD, that simulates the complex white-tailed deer-CWD system (13, 14). An important feature of this agent-based modeling framework is the ability to simulate age–sex-specific scenarios and interventions, as relevant individual host characteristics (age, sex, and group membership) and behaviors (dispersal, grouping behavior) have been incorporated in the constituent model programs. We illustrate how this model-based approach can be used to better understand phase-specific issues associated with CWD outbreaks. In this perspective, we argue that CWD management approaches should be phase specific (pre-establishment/transition/endemic) and should specifically take into consideration the need for pre-emptive, pre-detection strategies due to imperfect detection in the pre-establishment phase. A unique feature of CWD is the slow epidemic growth in the initial stage, and stochasticity plays an important role in determining whether the infection gets established in the deer population. Moreover, harvest strategies can influence the probability of CWD persistence. We conclude that a way forward should include the development of defensible pre-emptive harvest strategies that are locally relevant, sustainable and cost-effective, and prevent the establishment of CWD.

## Understanding CWD Dynamics

**Figure 1:**
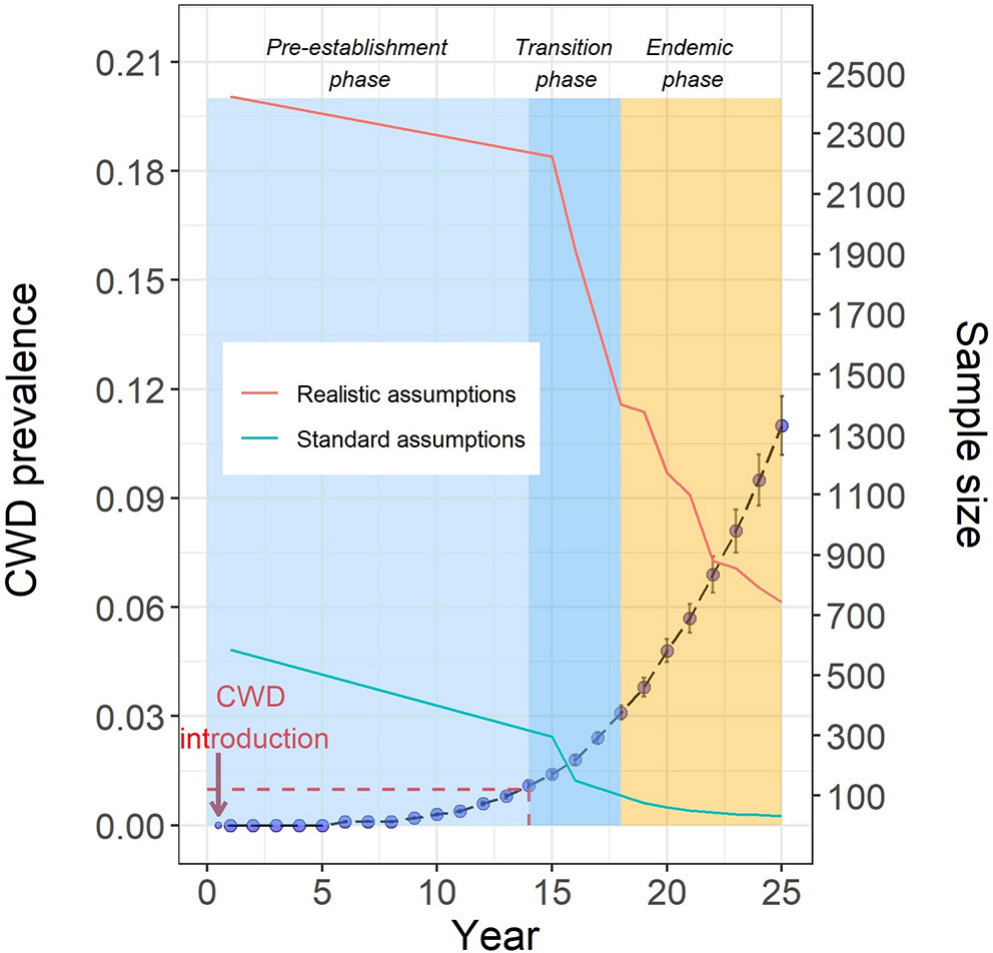
Blue solid circles represent the mean CWD prevalence (±SE) for iterations with active CWD cases. The model-derived CWD trajectory is divided in three phases: pre-establishment, transition, and endemic. Model-derived sample size requirements for CWD detection (turquoise line = standard assumptions, red line = realistic assumptions) are plotted along with the CWD outbreak trajectory. The y-axis on the right side is for sample size. For example, when the true prevalence of CWD in the model deer population is ~1% (year 14), the sample size needed for a 95% detection probability is 300, assuming a random distribution of CWD cases in the population and random sampling method (standard assumptions). For the same prevalence, but assuming a clustered distribution of CWD cases and non-random sampling (realistic assumptions), the sample size necessary for a 95% detection probability is ~2,200. Red dashed line highlights the pre-establishment phase features: prevalence below 1% for ~14 years post-introduction.

We derived CWD outbreak trajectories to provide a context for designing more efficient and sustainable CWD surveillance and management strategies. Model parameterization and implementation are detailed elsewhere (13). Here, we briefly describe the modeling process to derive outbreak trajectories. CWD was introduced in the model deer population (pre-harvest deer abundance ~52,800, representing a midwestern county landscape of ~721 square miles) by a dispersing yearling during the first year of the model run. The subsequent spread of CWD in the model deer population was documented over a 25 year period for each model simulation. Model output data from 100 iterations were summarized to generate a statistical portrait of CWD prevalence for each year of the model run (Figure 1; the dashed blue line represents CWD outbreak trajectory). CWD prevalence remains low (below 1%) for at least a decade after introduction, and this pattern is in agreement with field and modeling data from other studies in North America (15–17). Similar CWD outbreak patterns have been documented in white-tailed deer populations from Wisconsin, Pennsylvania and West Virginia. Such low prevalence rates after introduction have important implications for surveillance and management of CWD.

### The Issue of Sampling and Imperfect CWD Detection

In the early phase of the outbreak (~10–15 years after introduction into a natural population), detection of CWD using hunter-harvested deer (non-probabilistic sampling) is difficult, if not practically impossible, because the overall prevalence remains very low and cases are clustered, not randomly distributed (18–20). Nevertheless, at present, sample size calculations are usually undertaken without accounting for the clustering of cases and the non-probabilistic nature of hunter-harvest. As a result, the detection probability (or the confidence of detecting CWD) is likely overestimated (14, 21), and therefore, inferences about the presence or absence of CWD in the targeted population during the pre-establishment phase are unreliable (22, 23). Most often, by the time active surveillance detects CWD in a population, the disease is already well-established and difficult to eliminate (16, 24).

To highlight phase-specific sampling issues, we used the surveillance model (14) to calculate sample size targets for high detection probability (95%) over the course of the CWD outbreak simulating (a) current standard assumptions (randomly distributed CWD cases, random sampling) and (b) realistic assumptions (clustered CWD distribution and non-random sampling). Model-derived sample size requirements are plotted along with the CWD outbreak trajectory (Figure 1). Based on the model-derived insights about CWD spread dynamics and sampling requirements, we define three phases of CWD outbreak: (a) *pre-establishment* phase is the early stage of the outbreak characterized by low CWD prevalence (below 1%) and sample size requirements that are either difficult to achieve and unsustainable (using realistic assumptions) or unreliable (detection probability overestimated due to unrealistic assumptions); (b) *transition* phase follows the pre-establishment phase, characterized by an increasing prevalence (above 1%, but <3%) and a considerable decrease in the sample size requirement even with realistic assumptions; and (c) *endemic* phase characterized by rapidly increasing CWD prevalence and a corresponding decrease in sample size requirement for CWD detection using realistic assumptions. Thus, surveillance strategies at each phase necessitates different sample sizes to confidently detect the existence of CWD. Among other factors, environmental contamination likely plays an increasingly important role in CWD transmission in the endemic phase, further complicating disease management (3).

Here, we show that model-derived sample size requirements for confidently detecting CWD in the pre-establishment phase are considerably large if realistic assumptions are used (Figure 1). Moreover, the surveillance model as presented here assumes 100% test sensitivity. But the probability of obtaining a positive result when testing an infected individual (test sensitivity) is <100% which is of special relevance for CWD because test sensitivity is typically lower in the early infection stages of the disease (25). Therefore, the actual samples required for confidently detecting CWD will be likely larger than the model-derived sample size.

Other model approaches, such as Bayesian weighted surveillance (26) and risk-based scenario tree modeling (27, 28) have been proposed for the early detection of CWD and other emerging diseases. Utilizing targeted sampling of high–risk individuals may be more cost-effective and reduce the sample size needed to detect disease when compared to random or convenience sampling. However, regardless of the approach to determine sample sizes, CWD surveillance data will be biased if clustering of cases in the pre-establishment phase and very low prevalence are not accounted for. A modeling tool that incorporates spatial clustering of cases (like *Ov*CWD) will be particularly useful for determining realistic sample size requirements for confidently detecting CWD in the pre-establishment phase. Better still, such a tool can be used to set a realistic detection threshold to economize surveillance efforts for efficient management of CWD.

### Pre-establishment Phase CWD Dynamics and Pre-emptive Harvest Strategies

**Figure 2:**
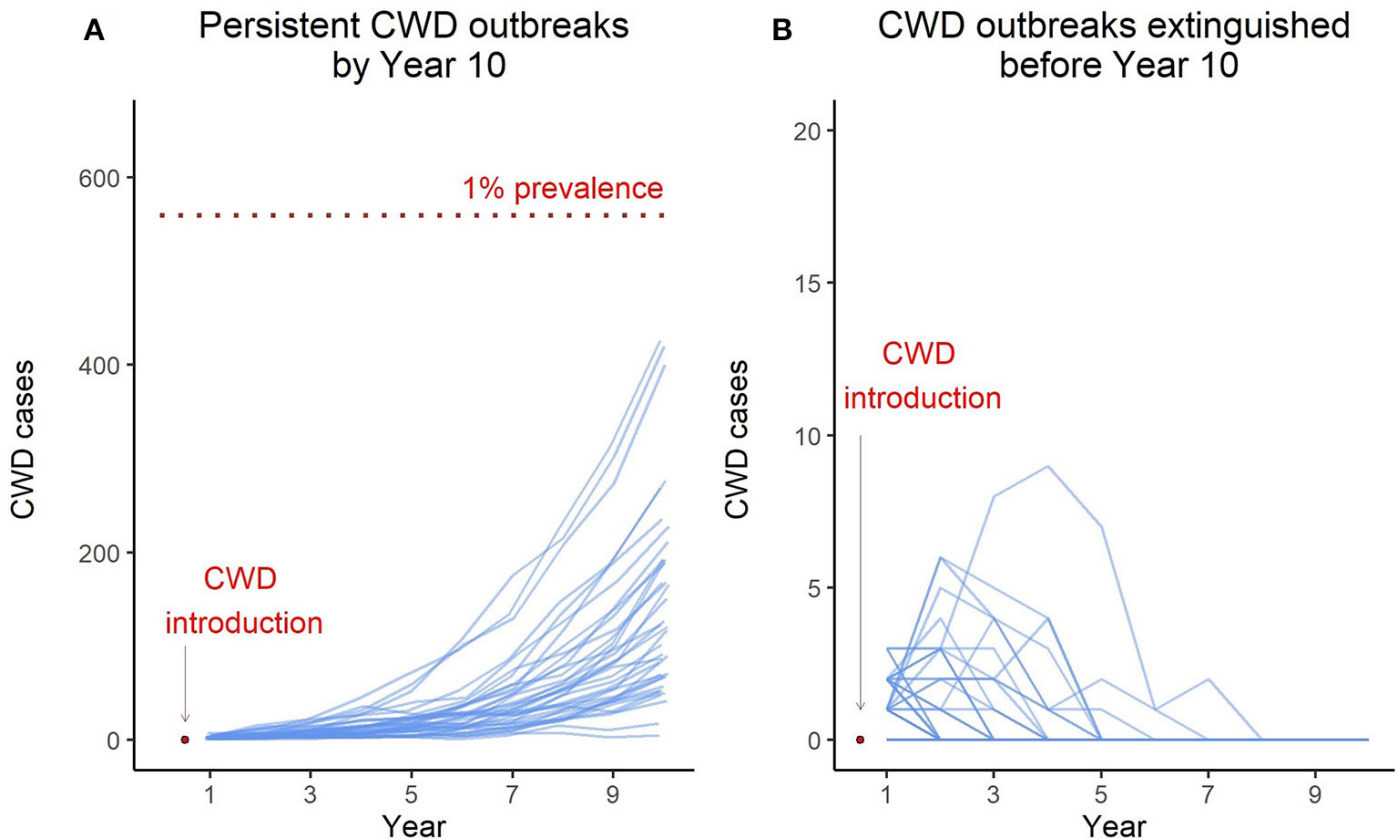
Blue lines in the left panel **(A)** represent model iterations where the CWD transmission persisted for 10 years. The red dotted line indicates 1% prevalence level. Blue lines in the right panel **(B)** represent model iterations where CWD was extinguished from the population before year 10. Note the y-axis scale for the right panel, the maximum number of cases in non-persistent outbreaks remained under 10.

CWD is difficult to detect in the early phase of the outbreak when prevalence is low, and difficult to eliminate in the later stage of the outbreak when CWD is established. Preventing widespread establishment of CWD in regional populations is the key to avoiding long-term population health and economic impacts caused by CWD. The early-stage CWD dynamics in the model deer population underscores an important feature that sets the context for pre-emptive management of CWD: every introduction event does not necessarily result in persistent CWD transmission in the population, and it is possible that multiple introduction events occur before CWD is eventually established. The probability that a CWD introduction event results in persistent CWD transmission in the population is underpinned by two stochastic processes occurring at the individual level: actual transmission of infection between an infected and susceptible individual and an infected individual surviving harvest mortality. *Ov*CWD explicitly simulates these stochastic processes, and therefore can be used to derive “CWD persistence probability” [calculated as the proportion of iterations that have active CWD transmission in year 10 post-introduction; this was referred to as CWD Outbreak probability by (13)]. For example, for the scenario described in this paper, CWD transmission persisted in 41 out of 100 iterations while CWD transmission was extinguished before year 10 in 59 iterations (Figure 2). Therefore, the CWD persistence probability for the model deer population under the current harvest regime is 0.41. This metric can be used to compare and contrast the efficacy of alternate management actions implemented in the early phase of CWD outbreak.

For a given region of interest, CWD transmission (and therefore CWD persistence probability) is influenced by multiple factors, including: habitat characteristics, host community (species-specific differences in transmission and susceptibility), genetic structure (e.g., PRNP variation), demographics, behavior (dispersal, migration, aggregation, etc.), population connectivity related to natural and man-made barriers, and, of course, harvest (19, 20, 29–34). Some of these features are difficult to manipulate on meaningful spatial and temporal scales whereas others, especially demographics, can be manipulated by altering harvest strategies so as to decrease the CWD persistence probability. Implementing such harvest strategies before CWD is detected in a region (therefore, pre-emptive strategies), with the objective of increasing the resilience of the deer population to the spread and persistence of CWD, could be an important step forward in the current fight against CWD.

## Discussion

The management of CWD shares challenges common for many wildlife diseases. A common limitation is the lack of a firm theoretic basis when managing wildlife diseases (35). We propose the use of *Ov*CWD as one possible solution in the case of harvest management of CWD. Phase-specific management actions have been advocated for wildlife diseases in general (36), and for CWD, is one clear recommendation arising both from our modeling and from earlier literature surveys (8). Yet, implementing actions sufficiently early is made difficult by the uncertainty about the current status of infection in areas where wildlife disease has not been detected (11). In the case of CWD, this uncertainty stems from logistical constraints on wildlife agencies' ability to match the sample size targets required for detection of CWD in the early phase of the outbreak. For this reason, a blanket surveillance strategy, without considering the phase of the outbreak, may be inherently appealing and politically popular but is inefficient and unsustainable. Furthermore, non-uniform sampling effort across regions complicates the assessment of CWD status in regional deer populations. What is needed is the ability to rapidly assess the status of CWD in areas where it has not been detected yet.

We have already illustrated the use of our model-based framework for guiding collection and analysis of surveillance data that relies on harvest-based sampling (14). It is nearly impossible to confidently confirm the absence of CWD in a population or to confidently detect CWD in the early phase of the outbreak. In our view, surveillance strategies for areas with uncertain CWD status should acknowledge inherent logistical and practical limitations and use a realistic disease detection threshold. As illustrated in this paper, large number of samples are required for confident detection of CWD in the *pre-establishment phase* compared to the *transition phase* (Figure 1). Therefore, instead of a difficult to achieve and unsustainable surveillance target, agencies should consider setting the detection threshold to coincide with the *transition phase* of the CWD outbreak (prevalence above 1%, but <3%). Moreover, the effect of non-random sampling is scale-dependent, and using smaller, ecologically based sampling units might reduce bias in probability of detection from non-random sampling and disease clustering (37).

From veterinary epidemiology, it is known that pre-emptive culling is required for diseases with latent stages or for those with initial low prevalence building up environmental reservoirs (38). Therefore, if CWD remains undetected with the detection threshold set for the transition phase, agencies should consider implementing pre-emptive harvest strategies tailored specifically for the target population. First, locally relevant harvest strategies that could be reasonably applied should be identified. These strategies will mostly involve manipulation of age and sex specific harvest rates, numbers removed, and spatial extent of affected area. The efficacy of such pre-emptive harvest strategies can then be evaluated using a simulation modeling framework like *Ov*CWD by deriving CWD persistence probabilities (13). Lower persistence probabilities can be interpreted as higher resiliency in the deer population to the spread and establishment of CWD. Additionally, CWD outbreak trajectories (and outbreak sizes) can be compared among different scenarios to assess their efficacy in limiting the spread of CWD. Wildlife agencies are likely to encounter resistance for pre-emptive strategies from stakeholders. Model-based simulations and virtual experiments can be used to communicate nuanced and complex management issues to stakeholders. Furthermore, user-friendly apps (Shiny apps, dashboard; e.g., https://rpubs.com/anyadoc/OvCWD_APR) can be developed to effectively illustrate these outcomes and ideas. Despite the publics' resistance that could occur with pre-emptive management strategies, such actions could help avoid much more invasive procedures such as population eradication.

Our modeling work highlights the importance of pre-emptive harvest strategies before CWD detection. By implementing model-evaluated harvest strategies pre-emptively, before CWD is detected in the population, wildlife agencies can improve their chances of reducing spread of CWD. Furthermore, management strategies implemented in the early stage of the outbreak during pre-introduction or pre-establishment will be relatively sustainable and logistically doable. Such pre-emptive strategies would be more palatable to the North American public than eradicating entire populations [e.g., (39)].

## Data Availability Statement

Publicly available datasets were analyzed in this study. This data can be found here: https://github.com/anyadoc/proactiveCWDmgmt. Model codes are openly available for download here: MIOvPOP (Version 1.1.0) https://doi.org/10.25937/kv07-3e08; MIOvCWD (Version 1.0.0) https://doi.org/10.25937/6qeq-1c13; MIOvPOPsurveillance (Version 1.0.0) https://doi.org/10.25937/fdke-rp28.

## Author Contributions

AB: conceptualization, model simulation and model output analysis, writing—original draft, writing-review and editing, prepared, and reviewed the manuscript. JJM, JRM, JS, HV, and AM: conceptualization and writing-review and editing. All authors contributed to the article and approved the submitted version.

## Conflict of Interest

The authors declare that the research was conducted in the absence of any commercial or financial relationships that could be construed as a potential conflict of interest.

## References

[1] Edmunds DR Kauffman MJ Schumaker BA Lindzey FG Cook WE Kreeger TJ Chronic wasting disease drives population decline of white-tailed deer. PLoS ONE. (2016) 11:e0161127 10.1371/journal.pone.0161127 27575545 PMC5004924

[2] DeVivo MT Edmunds DR Kauffman MJ Schumaker BA Binfet J Kreeger TJ. Endemic chronic wasting disease causes mule deer population decline in Wyoming. PLoS ONE. (2017) 12:e0186512. 10.1371/journal.pone.0186512 29049389 PMC5648191

[3] Almberg ES Cross PC Johnson CJ Heisey DM Richards BJ. Modeling routes of chronic wasting disease transmission: environmental prion persistence promotes deer population decline and extinction. PLoS ONE. (2011) 6:e19896. 10.1371/journal.pone.0019896 21603638 PMC3094393

[4] Bishop RC The economic impacts of Chronic Wasting Disease (CWD) in Wisconsin. Hum Dimens Wildl. (2004) 9:181–92. 10.1080/10871200490479963

[5] Rivera NA Brandt AL Novakofski JE Mateus-Pinilla NE. Chronic wasting disease in cervids: prevalence, impact and management strategies. Vet Med Res Rep. (2019) 10:123–39. 10.2147/VMRR.S197404 31632898 PMC6778748

[6] Hewitt DG Hunters and the conservation and management of white-tailed deer (*Odocoileus virginianus*). Int J Environ Stud. (2015) 72:839–49. 10.1080/00207233.2015.1073473

[7] Macaulay L The role of wildlife-associated recreation in private land use and conservation: providing the missing baseline. Land Use Policy. (2016) 58:218–33. 10.1016/j.landusepol.2016.06.024

[8] Uehlinger FD Johnston AC Bollinger TK Waldner CL. Systematic review of management strategies to control chronic wasting disease in wild deer populations in North America. BMC Vet Res. (2016) 12:173. 10.1186/s12917-016-0804-7 27549119 PMC4994292

[9] Holsman RH Petchenik J Cooney EE CWD After “the Fire”: six reasons why hunters resisted Wisconsin's eradication effort. Hum Dimens Wildl. (2010) 15:180–93. 10.1080/10871201003718029

[10] Mysterud A Strand O Rolandsen CM Efficacy of recreational hunters and marksmen for host culling to combat chronic wasting disease in reindeer. Wildl Soc Bull. (2019) 43:683–92. 10.1002/wsb.1024

[11] Grant EHC Muths E Katz RA Canessa S Adams MJ Ballard JR Using decision analysis to support proactive management of emerging infectious wildlife diseases. Front Ecol Environ. (2017) 15:214–21. 10.1002/fee.1481

[12] Winter SN Escobar LE. Chronic wasting disease modeling: an overview. J Wildl Dis. (2020) 56:741–58. 10.7589/2019-08-213 32544029

[13] Belsare A Stewart C OvCWD: an agent-based modeling framework for informing chronic wasting disease management in white-tailed deer populations. Ecol Solut Evid. (2020) 1:e12017 10.1002/2688-8319.12017

[14] Belsare A Gompper M Keller B Sumners J Hansen L Millspaugh J. An agent-based framework for improving wildlife disease surveillance: a case study of chronic wasting disease in Missouri white-tailed deer. Ecol Modell. (2020) 417:108919. 10.1016/j.ecolmodel.2019.108919 32189826 PMC7079769

[15] Miller MW Williams ES McCarty CW Spraker TR Kreeger TJ Larsen CT. Epizootiology of chronic wasting disease in free-ranging cervids in Colorado and Wyoming. J Wildl Dis. (2000) 36:676–90. 10.7589/0090-3558-36.4.676 11085429

[16] Jennelle CS Henaux V Wasserberg G Thiagarajan B Rolley RE Samuel MD. Transmission of chronic wasting disease in wisconsin white-tailed deer: implications for disease spread and management. PLoS ONE. (2014) 9:e91043. 10.1371/journal.pone.0091043 24658535 PMC3962341

[17] EFSA Panel on Biological Hazards (BIOHAZ) Ricci A Allende A Bolton D Chemaly M Davies R Fernández Escámez PS. Scientific opinion on chronic wasting disease (II). EFSA J. (2018) 16:e05132. 10.2903/j.efsa.2018.5132 32625679 PMC7328883

[18] Walsh DP Enhanced Surveillance Strategies for Detecting and Monitoring Chronic Wasting Disease in Free-Ranging Cervids. U.S. Geological Survey Open-File Report (2012). Available online at: http://pubs.usgs.gov/of/2012/1036/

[19] Joly DO Samuel MD Langenberg JA Blanchong JA Batha CA Rolley RE. Spatial epidemiology of chronic wasting disease in Wisconsin white-tailed deer. J Wildl Dis. (2006) 42:578–88. 10.7589/0090-3558-42.3.578 17092889

[20] Osnas EE Heisey DM Rolley RE Samuel MD. Spatial and temporal patterns of chronic wasting disease: fine-scale mapping of a wildlife epidemic in Wisconsin. Ecol Appl. (2009) 19:1311–22. 10.1890/08-0578.1 19688937

[21] Samuel MD Joly DO Wild MA Wright SD Otis DL Werge RW Surveillance Strategies for Detecting Chronic Wasting Disease in Free-Ranging Deer and Elk: Results of a CWD Surveillance Workshop, 10-12 December 2002. Madison, WI: United States Geological Survey National Wildlife Health Center (2003).

[22] Rees EE Merrill EH Bollinger TK Hwang YX Pybus MJ Coltman DW. Targeting the detection of chronic wasting disease using the hunter harvest during early phases of an outbreak in Saskatchewan, Canada. Prev Vet Med. (2012) 104:149–59. 10.1016/j.prevetmed.2011.10.016 22137503

[23] Nusser SM Clark WR Otis DL Huang L Sampling considerations for disease surveillance in wildlife populations. J Wildl Manage. (2008) 72:52–60. 10.2193/2007-317

[24] Miller MW Fischer JR The first five (or more) decades of chronic wasting disease: lessons for the five decades to come. in Transactions of the 81st North American Wildlife and Natural Resources Conference Pittsburg, PA, 110–120.

[25] Viljugrein H Hopp P Benestad SL Nilsen EB Våge J Tavornpanich S A method that accounts for differential detectability in mixed samples of long-term infections with applications to the case of chronic wasting disease in cervids. Methods Ecol Evol. (2019) 10:134–45. 10.1111/2041-210X.13088

[26] Jennelle CS Walsh DP Samuel MD Osnas EE Rolley R Langenberg J Applying a Bayesian weighted surveillance approach to detect chronic wasting disease in white-tailed deer. J Appl Ecol. (2018) 55:2944–53. 10.1111/1365-2664.13178

[27] Hadorn DC Stärk KD. Evaluation and optimization of surveillance systems for rare and emerging infectious diseases. Vet Res. (2008) 39:57. 10.1051/vetres:2008033 18651991

[28] Mysterud A Hopp P Alvseike KR Benestad SL Nilsen EB Rolandsen CM. Hunting strategies to increase detection of chronic wasting disease in cervids. Nat Commun. (2020) 11:4392. 10.1038/s41467-020-18229-7 32873810 PMC7463264

[29] Conner MM Miller MW Movement patterns and spatial epidemiology of a prion disease in mule deer population units. Ecol Appl. (2004) 14:1870–81. 10.1890/03-5309

[30] Miller MW Hobbs NT Tavener SJ. Dynamics of Prion disease transmission in Mule deer. Ecol Appl. (2006) 16:2208–14. 10.1890/1051-0761(2006)016-2208:DOPDTI-2.0.CO 17205898

[31] Blanchong JA Samuel MD Scribner KT Weckworth B Langenberg JA Filcek KB. Landscape genetics and the spatial distribution of chronic wasting disease. Biol Lett. (2008) 4:130–3. 10.1098/rsbl.2007.0523 18077240 PMC2412942

[32] Heisey DM Osnas EE Cross PC Joly DO Langenberg JA Miller MW Linking process to pattern: estimating spatiotemporal dynamics of a wildlife epidemic from cross-sectional data. Ecol Monogr. (2010) 80:221–40. 10.1890/09-0052.1

[33] Samuel MD Storm DJ. Chronic wasting disease in white-tailed deer: infection, mortality and implications for heterogeneous transmission. Ecology. (2016) 97:3195–205. 10.1002/ecy.1538 27870037

[34] Miller MW Runge JP Holland AA Eckert MD Hunting pressure modulates prion infection risk in mule deer herds. J Wildl Dis. (2020) 56:781–90. 10.7589/JWD-D-20-00054 33600602

[35] Joseph MB Mihaljevic JR Arellano AL Kueneman JG Preston DL Cross PC. Taming wildlife disease: bridging the gap between science and management. J Appl Ecol. (2013) 50:702–12. 10.1111/1365-2664.12084 32336775 PMC7166616

[36] Langwig KE Voyles J Wilber MQ Frick WF Murray KA Bolker BM Context-dependent conservation responses to emerging wildlife diseases. Front Ecol Environ. (2015) 13:195–202. 10.1890/140241

[37] Joly DO Samuel MD Langenberg JA Rolley RE Keane DP. Surveillance to detect chronic wasting disease in white-tailed deer in Wisconsin. J Wildl Dis. (2009) 45:989–97. 10.7589/0090-3558-45.4.989 19901375

[38] Tildesley MJ Bessell PR Keeling MJ Woolhouse MEJ. The role of pre-emptive culling in the control of foot-and-mouth disease. Proc R Soc B Biol Sci. (2009) 276:3239–48. 10.1098/rspb.2009.0427 19570791 PMC2817163

[39] Mysterud A Rolandsen CM. A reindeer cull to prevent chronic wasting disease in Europe. Nat Ecol Evol. (2018) 2:1343–5. 10.1038/s41559-018-0616-1 29988166

